# The application of poorly crystalline silicotitanate in production of ^225^Ac

**DOI:** 10.1038/s41598-019-48021-7

**Published:** 2019-08-14

**Authors:** Jonathan Fitzsimmons, Alyson Abraham, Demetra Catalano, Ali Younes, Cathy S. Cutler, Dmitri Medvedev

**Affiliations:** 1 Medical Isotope Research & Production Laboratory, Collider-Accelerator Division, Brookhaven National Laboratory, Upton, NY 11973 USA; 2 Chemistry Department, Stony Brook University, Stony Brook, NY 11794 USA; 3 Biochemistry Department, Stony Brook University, Stony Brook, NY 11794 USA

**Keywords:** Analytical chemistry, Nuclear chemistry, Inorganic chemistry, Process chemistry, Radiotherapy

## Abstract

Actinium-225 (^225^Ac) can be produced from a Thorium-229/Radium-225 (^229^Th/^225^Ra) generator, from high/low energy proton irradiated natural Thorium or Radium-226 target. Titanium based ion exchanger were evaluated for purification of ^225^Ac. Poorly crystalline silicotitanate (PCST) ion exchanger had high selectivity for Ba, Ag and Th. ^225^Ac was received with trace amounts of ^227^Ac, ^227^Th and ^223^Ra, and the solution was used to evaluate the retention of the isotopes on PCST ion exchanger. Over 90% of the ^225^Ac was recovered from PCST, and the radiopurity was >99% (calculated based on ^225^Ac, ^227^Th, and ^223^Ra). The capacity of the PCST inorganic ion exchange for Barium and ^232^Th was determined to be 24.19 mg/mL for Barium and 5.05 mg/mL for Thorium. PCST ion exchanger could separate ^225^Ac from isotopes of Ra and Th, and the process represents an interesting one step separation that could be used in an ^225^Ac generator from ^225^Ra and/or ^229^Th. Capacity studies indicated PCST could be used to separate ^225^Ac produced on small ^226^Ra targets (0.3–1 g), but PCST did not have a high enough capacity for production scale Th targets (50–100 g).

## Introduction

Ion-exchange chromatography has been successfully used to separate radioisotopes for medical applications, nuclear fuel reprocessing and other applications^1,2^. However in many instances the ion-exchange material lacks desired selectivity. Current methods of separation rely on commercially available ion-exchange resins that preferentially bind the element based on charge^3^. Extraction chromatography methods have been developed for some separations^4^, but the extraction chromatography resins sometime have slow flow rates, and the extractant can be eluted. Often the process to purify a radioisotope requires the use of multiple columns and result in consuming more time and labor. Chemical separations used in accelerator isotope production process at Brookhaven National Lab (BNL) present interesting challenges. The target masses for production scale targets irradiated at BNL are over 50 grams, and the mass of the radioisotopes produced is less than 0.0001 grams^5,6^. The separation presents challenges if no ion exchange resins are available that have more selective for the isotope of interest rather than the target material.

Production of ^225^Ac has been of particular interest recently since efficacy of this material has been demonstrated in a treatment of certain types of cancer. ^225^Ac can be made available by several routes: separation from ^229^Th/^225^Ra generator, by high energy (100–200 MeV) proton irradiation of natural Thorium target, or by irradiation ^226^Ra target with low energy proton (10–24 MeV)^7,8^. Chemical separation of ^225^Ac from thorium irradiated with high energy protons is especially challenging. The irradiation results in fission of the thorium in the target and, in addition to ^225^Ac, produces a variety of potentially useful radionuclides such as ^111^Ag, ^105^Rh, ^140^La, Ra isotopes, and ^140^Ba. As previously mentioned, ^225^Ac (t_1/2_ = 9.9 days) and its daughter; ^213^Bi (t_1/2_ = 45 min) are emerging as important isotope for targeted alpha therapy^9–11^. Other isotopes in the list can be used for beta therapy (^111^Ag, ^105^Rh, and ^140^La), or as a parent in medical isotope generators, for example Radium isotopes and ^140^Ba.

The use of inorganic ion-exchange materials which selectivity stems from the crystal structure of the ion-exchanger could provide a more attractive mode of separation. Crystalline silicotitanate (CST) and the poorly crystalline CST (PCST) have been synthesized hydrothermally in alkaline media^12^. The reduced crystallinity was obtained by either shortening the reaction time at the same synthesis temperature for CST (200 °C) or reducing the temperature to 170 °C and changing the hydrothermal reaction time. The CST inorganic ion exchangers were initially developed in the 1960s and have been evaluated for nuclear waste treatment due to the materials remarkable selectivity toward Cs and Sr^13–15^. Studies showed that Cs and Sr^2+^ cations demonstrate higher rate of uptake by poorly crystalline CST compared to the crystalline form. This was attributed to the higher surface area and smaller particle size, which was highlighted to account for the increased rate even though there would be otherwise slow diffusion through the channels. The high selectivity of CST ion exchanger for Cs and Sr was used to decontaminate the Fukushima site^16^.

Titanium-based ion exchange materials have been demonstrated to be selective for strontium and actinides in highly alkaline environments^17^. The studies reported herein seek to synthesize, characterize, and evaluate PCST inorganic ion exchanger in the separation process of ^225^Ac from irradiated Th target. During the accelerator production of ^225^Ac from a ^232^Th target ^227^Ac and the daughters of ^227^Ac are coproduced^18^. The daughters of ^227^Ac (^227^Th and ^223^Ra) can be separated from ^225^Ac during the purification process^19^. However after purification the ^227^Ac would grow in and reduce the purity of ^225^Ac^18^. Various literature studies to purify ^225^Ac from Thorium and/or radium have focused on organic cation or anion ion exchange resins^1,2^. The PCST ion exchanger has been synthesized and evaluated for the removal of Sr, Cs and other isotopes from waste streams^20^. In this manuscript the purification of ^225^Ac from ^227^Th and ^223^Ra with PCST ion exchanger was developed. The utility of the purification process was evaluated in the following applications: ^225^Ac production from proton irradiated Th or Ra, ^225^Ac produced from the ^229^Th/^225^Ra generator, and to purify ^225^Ac from the daughters of ^227^Ac.

## Results

**Figure 1:**
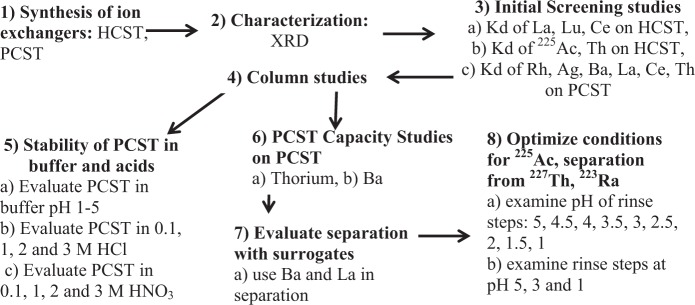
Flow chart illustrating the sequence of studies reported in the manuscript.

Inorganic ion-exchangers were synthesized by hydrothermal synthesis, purified and the phases were confirmed by comparing XRD patterns to published results^20^. Figure 1 outlines the study design to evaluate the inorganic ion exchangers. Initially, ion-exchange properties of the synthesized materials were evaluated by the batch method, and distribution coefficients (K_d_) were determined for ^225^Ac and Th. Subsequent studies determined the K_d_ of several elements (Th, La, Ce, Rh, Ag, and Ba) on PCST ion exchanger. Optimal conditions for the separation on PCST inorganic ion-exchanger were evaluated with a representative sample containing ^225^Ac, ^227^Th, and ^223^Ra. Capacity studies were performed with barium and thorium on PCST to determine if the ion exchanger could be used for radium (0.3 g) and/or thorium (50–100 g) production targets. The stability of the PCST ion exchanger was determined in: ammonium acetate buffer from pH 5 to 1, hydrochloric or nitric acid at 0.1,1, 2 and 3 M.

### Evaluation of inorganic Ion-Exchanger Selectivity

**Figure 2:**
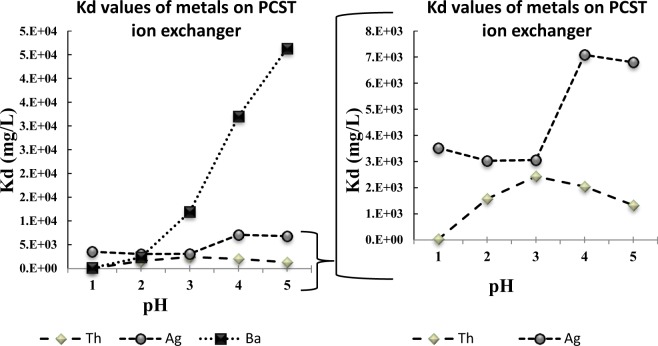
Distribution coefficients (Kd) of Ba, Th, and Ag on PCST ion exchanger at pH values from 1 to 5 in 0.5 M ammonium acetate. La, Ce, and Rh had Kd values less than 100 mg/L.

The evaluation of the selectivity of poorly crystalline silicotitanate (PCST) for Rh, Ba, La, Ce, Th, and Ag were performed with batch studies, and the data for Th, Ag, and Ba are presented in Fig. 2. The results of the PCST ion exchanger show an insignificant selectively at low pH for Ba, La, Ce, Th and Rh, with a higher selectivity for Ag (3465 ml/g) at a pH of 1. As the pH goes from 1 to 5 the PCST ion exchanger increases selectivity for Ba, and Ag (46305 ml/g, 6796 ml/g respectively). The PCST ion exchanger had low selectivity for Ce, La and Rh (100 ml/g, 71 ml/g and 85 ml/g respectively). The distribution coefficients for the trivalent cations Ce and La increased as the pH went 1 to 5.

### Column studies

To increase the flow rates buffer was added to the 100–200 mesh PCST material and the solution was decanted. This was able to remove fine particles of PCST material, and the flow rates increased to 0.25–1 ml/min.

#### PCST breakdown study

**Figure 3:**
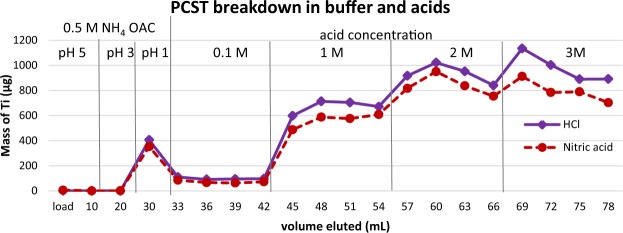
Acid study examining the breakdown of PCST in the presence of various concentrations of 0.5 M ammonium acetate and hydrochloric or nitric acid. 10 mL (20 bed volumes) was used for the 0.5 M ammonium acetate load and rinse steps then each acid concentration was rinsed with 4 × 3 mL rinses.

The quantification limit for Ti on the ICP-OES was defined by analysis of diluted standards and the acceptance criteria for the true concentration value and the % RSD was within 10%. The quantification limit for Ti by ICP-OES was determined to be 0.005 ppm. ICP-OES analysis of all samples indicated Ti breakthrough (Fig. 3). The Ti breakthrough was lowest in 0.5 M ammonium acetate pH 5 with 0.05–6 µg of Ti present in the load, 0.5 M ammonium acetate pH 5 and 3 rinses. Rinsing the PCST material with ammonium acetate at pH 1 resulted in 352–405 µg of Ti. In subsequent rinses with 0.1 M HCl or nitric acid the PCST material showed a slight higher amount of Ti breakthrough in HCl (91–110 µg) versus nitric acid (63–86 µg). Higher concentrations of acid resulted in more breakthrough of the Ti from the PCST with 1 M (480–700 µg Ti per fraction) 2 M (750–1022 µg Ti per fraction) and 3 M (703–1135 µg Ti per fraction). In all elutions with HCl or nitric acid breakthrough of the Ti from the PCST material was higher for HCl.

#### Separations of Th, ^225^Ac and other metals

In summary the Kd data indicated: the PCST ion exchanged has favorable properties to capture Thorium at pH values of 1 to 2 while ^225^Ac would be less favorable; the resin has high affinity for Ba at pH values of 3 to 5, and is more selective for Ag at pH values of 1, 4 or 5 than Th, La, Ce, and Rh. A column of PCST ion exchanger was used to capture Thorium and barium from a solution containing Th, Ba, ^225^Ac, Rh, La, Ag, and Ce in 0.5 M NaOAc at pH 2 (see Supplementary Fig. S4). The eluted solution contained 95% of Ac, 90% of Ce, 73% of La and 77% of Rh. The data indicates 15% of La, 5.8% of Rh, and Th, Ag and Ba were totally absorbed and retained on the PCST column. The absorbed metals except Ag were recovered from the PCST column using 3 M nitric acid solution. The capacity of the PCST inorganic ion exchange materials for Barium and ^232^Th was determined to be 24.19 mg/mL for Barium and 5.05 mg/mL for Thorium.

#### Ba and La column studies

The PCST ion exchanger was able to retain Ba in 0.5 M ammonium acetate at pH 5 while eluting La (see Supplementary Fig. S2). Combining the load and rinse 1–3 provided 85% recovery of La while only 0.15% of the Ba was present. Rinse solutions 4–5 and elution solutions 1–2 recovered 99.8% of the barium with 92.8% of the barium eluting in 0.5 M ammonium acetate at pH 1.

#### ^223^Ra, ^225^Ac, ^227^Th studies

**Figure 4:**
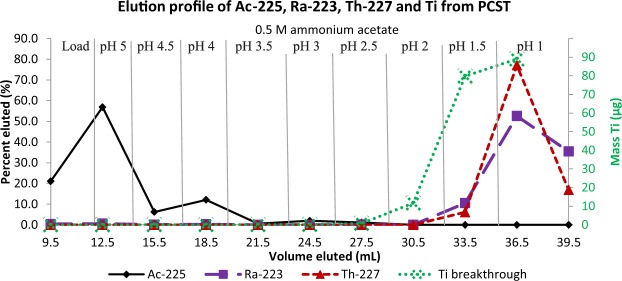
pH studies: A Separation of ^225^Ac, ^223^Ra and ^227^Th using a PCST ion exchanger in 0.5 M ammonium acetate. ^223^Ra and ^227^Th and ^225^Ac is retained at pH values greater than 3. ^223^Ra and ^227^Th are eluted at more acidic pH values. Titanium present in fractions associated with study A, illustrating the breakdown of PCST material at lower pH (in green).

pH study & PCST breakdown: A study was performed with PCST material with ^225^Ac, ^223^Ra and ^227^Th in 0.5 M ammonium acetate at pH 5 and the column was rinsed with the buffer at 4.5, 4, 3.5, 3, 2.5, 2.0, 2.5, 1.5, 1 (Fig. 4). The ^225^Ac eluted with two peaks at pH 5 and pH 3 with 96% eluting in the load and fractions with a pH from 5–3.5. The ^223^Ra and ^227^Th were both retained on the column and began to elute from the column at pH 1.5 with 98–100 percent of the isotopes eluting in the buffer at pH 3 to 1. The activity retained on the column was not measured. The breakdown of the PCST was evaluated and Ti was below quantification limits in the eluted load and pH 5 solutions, but Ti was quantified in all other fractions. The amount of Ti breakthrough in buffer at pH values from 4.5–2.5 was less than 1 μg per fraction. Eluting the PCST column with 0.5 M ammonium acetate buffer at pH 2, 1.5 and 1 resulted in higher breakthrough of Ti (11.7, 80, and 89 μg).

Initial optimization of the separation with PCST: Initial optimization of the separation examined using a rinse sequences with 6 bed volumes (BV) or column volume of 0.5 M ammonium acetate at both pH 5 and 3, and 12 BV at pH 1 (see Supplementary Fig. S3). Combining the load, pH 5 and 3 rinses resulted in 91% of the ^225^Ac, and approximately 39–41% of the ^225^Ac was eluted in pH 5 and 34–42% of the ^225^Ac was eluted in the pH 3 rinse. The 0.5 M ammonium acetate pH 1 rinse step eluted 44–81% of the Ra and 95% of the Thorium. The column retained 0.1–1.4% of the ^225^Ac and 19–56% of the ^223^Ra.

**Figure 5:**
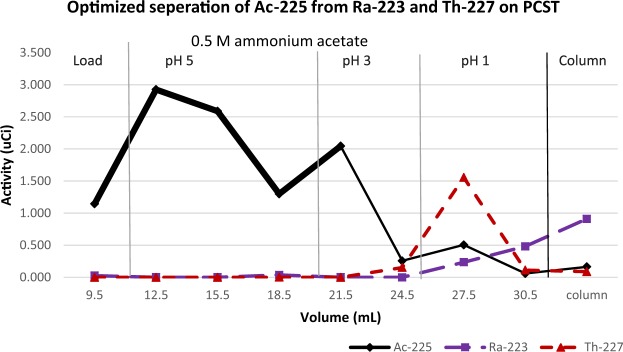
Optimized separation of ^225^Ac, ^223^Ra and ^227^Th using a PCST ion exchanger in 0.5 M ammonium acetate. ^225^Ac solution containing ^223^Ra and ^227^Th using a 0.5 mL column of PCST ion exchange material. The load, pH 5 and the first pH 3 rinse (heavy black line) contained 91% of ^225^Ac with a radiopurity of 99.3%. The second pH 3 rinse and pH 1 rinses eluted 95% of ^227^Th. The pH 1 rinse eluted 42% of ^223^Ra and 53.7% was retained on the column.

Optimized purification and PCST breakthrough: The rinse sequence used: 3X3BV of 0.5 M ammonium acetate at pH 5, 2X3BV of the buffer at pH 3, 2X3 BV of the buffer at pH 1 (Fig. 5). Combining the load, pH 5 and the first pH 3 rinse (heavy black line) resulted in the elution of 91% of the ^225^Ac. Based on the activity of ^225^Ac, ^223^Ra and ^227^Th the initially radionuclidic purity of the ^225^Ac was 78.4%, and the radionuclidic purity of the purified ^225^Ac in the combined fractions was 99.3%. In the pH 5 rinse step 62.9% of the ^225^Ac was eluted and in the pH 3 rinses 21.3% of the ^225^Ac was eluted with 18.9% eluting in the first pH 3 rinse and only 2.3% eluted in the second pH 3 rinse step. The second pH 3 rinse and the pH 1 rinses eluted 95% of ^227^Th. The pH 1 rinse contained 42% of ^223^Ra and 53.7% was retained on the column. Ti breakthrough was checked in the second pH 5, first pH 3 and both pH 1 fractions, and the Ti breakthrough was 0.025, 0.29, 120.7 and 142.5 µg per fraction consistent with previous studies.

## Discussion

Different separation methods and materials are being evaluated to purify ^225^Ac from Thorium, so that the US Department of Energy can supply ^225^Ac to researchers and clinicians on clinical scales^19,21–23^. In this manuscript PCST ion-exchanger was evaluated to determine if the material can be utilized for the purification of ^225^Ac from Thorium or radium targets or in a ^229^Th/^225^Ra generator.

### Column studies

Column studies with PCST ion exchanger were plagued with poor (1 ml/15 min) or no flow rates. Sieving the PCST material through various mesh filters did little to increase the flow rates. In all column studies 100–200 mesh PCST was used and reasonable flow rates were achieved by soaking the PCST in buffer and decanting the buffer.

#### Acid study of PCST

Ammonium acetate buffer at pH 1 and both hydrochloric acid and nitric acid at a concentration of 0.1 M lead to breakdown of PCST material. The breakdown of the PCST at pH 5–3 is far less then at more acidic pH values. Using the PCST material at pH 1 or in 1, 2, and/or 3 M acid may require a cleanup column to remove Ti breakthrough.

#### Separation of Ba from La

The Ba-La separation was conducted to assess the potential of a separation of Ra and Ac radioisotopes with a PCST column, with the stable isotopes serving as surrogates for the radioisotopes. The elution of both metals at separate pH values was clear, and La eluted at pH 5 while Ba eluted at pH 1 (see Supplementary Fig. S2). Application: This purification approach could be used to separate ^140^La from ^140^Ba in a generator system. The study indicates all trivalent lanthanides will be eluted at pH 5 with ^140^La and ^225^Ac.

#### Evaluating PCST ion exchangers for purification of ^225^Ac

The elution of Ba and La on PCST was repeated with ^223^Ra, ^227^Th and ^225^Ac, and the separation was optimized to purify ^225^Ac.

The separation of ^225^Ac from ^223^Ra and ^227^Th had good reproducibility. In five studies (the pH study, three optimization studies with ^225^Ac, ^223^Ra and ^227^Th, and the column studies ^225^Ac, Th, Ag, Ba, Rh, Ce, La) greater than 90% of the ^225^Ac eluted in the load, pH 5 and/or pH 3 solutions. The optimized elution method to separate ^225^Ac was selected based on the elution of the highest percentage of ^225^Ac with the lowest percentage of impurities, in this case, ^223^Ra and ^227^Th. The optimized method rinsed the PCST column with more 0.5 M ammonium Acetate at pH 5 and 3 resulted in a shift in the ^225^Ac elution peak. The result was more ^225^Ac was eluted earlier with 92% eluted in the pH 5 and first pH 3 rinse step. The process produced very pure ^225^Ac with ^223^Ra and ^227^Th eluting at low pH, and this data indicates the optimized method with PCST material could be used in several different production approaches to purify ^225^Ac from Thorium and/or Radium radioisotopes.

### Applications of separation

^225^Ac from a ^225^Ra/^229^Th generator: ^225^Ac has been produced at Oak Ridge National Lab (ORNL) from a ^225^Ra/^229^Th generator and they produce 5.5 × 10^10^ Bq (~1.5 Ci) per year^24^. The one column separation of ^225^Ac from ^223^Ra and ^227^Th with PCST could simplify the multicolumn approach used at ORNL to purify ^225^Ac in the ^225^Ra/^229^Th generator. The ORNL process is a four step chemical process with two MP1/NO_3_ columns to separate ^225^Ac and ^225^Ra from ^229^Th. Then the ^225^Ac is purified from ^225^Ra with two AG50X4/1.2 HNO_3_ purification steps. The high selectivity of PCST for both thorium and radium would simplify the purification of ^225^Ac, and the process would be one column, making the purification shorter than the ORNL process. Accelerator produced ^225^Ac: Accelerator produced ^225^Ac can be produced at high energy (>100 MeV) with a natural thorium target or at low energy (10–25 MeV) from a ^226^Ra target. PCST for the purification of ^225^Ac from ^232^Th targets: Although the PCST column worked to separate ^225^Ac from Thorium, Ra and some fission products the approach is similar to published separations that capture Thorium on an MP1 column and let ^225^Ac pass through the column^24^. This separation strategy would work for smaller thorium target. For larger scale clinical production of ^225^Ac with 50 to 100 g Thorium targets and potential Thorium stack targets could be required resulting in 100–600 grams of Thorium in the separation. The small capacity of PCST for Thorium indicates a large mass of PCST ion exchanger would be needed to capture all the Thorium. To process one 50 gr Thorium target it was estimated 10 L PCST column would be needed, so the material does not have a reasonable capacity to purify ^225^Ac from Thorium targets. PCST for the purification of ^225^Ac from ^226^Ra targets: Low energy protons irradiating a 0.3 g ^226^Ra target can produce clinical scales of ^225^Ac (~ 1 Ci/target)^7,25^. The amount of PCST ion exchange material to process a ^226^Ra target was estimated from capacity studies with barium, and the data indicates a minimum of a 12.5 mL PCST column would be needed to retain ^226^Ra in the target. The PCST separation method to purify ^225^Ac could be used to purify ^225^Ac from a ^223^Ra targets. ^226^Ra could be eluted from the PCST ion exchanger in pH 1 buffer, and the process could be used to recycle the ^226^Ra.

Removal of radio impurities (^223^Ra and ^227^Th) in accelerator produced ^225^Ac from Th targets: Accelerator produced ^225^Ac from a proton irradiated thorium targets has 0.1% abundance of ^227^Ac (t_1/2_ = 21.8 years) at end of bombardment. ^227^Ac decays to ^223^Ra (t_1/2_ = 11.4 days) and ^227^Th (t_1/2_ = 18.5 days), and ^227^Ac and the daughters represent the major radio- impurities for ^225^Ac. The expiration of a batch of accelerator ^225^Ac is defined by the purified ^225^Ac failing one of its specifications, which are currently being determined by the Trilab ^225^Ac team. The radiopurity of ^225^Ac will likely be the first specification that fails. The Trilab team has estimated the radiopurity post purification in the presence of ^227^Ac and ^227^Ac and daughters for BLIP produced ^225^Ac from Th targets^18^. Removal of ^223^Ra and ^227^Th would increase the radiopurity of ^225^Ac produced from a Th target at BLIP. During the development of the PCST separation the original radiopurity of ^225^Ac was 78.4% (calculated from ^225^Ac, ^227^Th and ^223^Ra), but after performing the purification with PCST column the radiopurity was 99.3% and the recovery of ^225^Ac was 92.4%. This improvement in radiopurity indicates that this separation can be used to extend ^225^Ac shelf life by removing ^227^Ac daughters, ^223^Ra and ^227^Th. A PCST column run on the ^225^Ac sample would remove ^223^Ra and ^227^Th; increasing the radiopurity to greater than 98% and this could be done out to 30.1 days. For ^225^Ac produced from a Th target at BLIP the radiopurity falls below 98% after 14.9 days after the last separation step. A radiopurity greater than 98% can be achieved with a PCST column out to 27.8 days. In a clinical setting, the collection of the 18 BV of the pH 5 rinse and first 6 BV of the pH 3 rinse would recover 92.4% of eluted ^225^Ac and a negligible percentage of impurities. This 12 mL sample of ^225^Ac would be easy to evaporate.

## Conclusion

The impact of developing a material with specific isotope and/or metal selectivity would potentially be invaluable in assisting with efforts in medical isotope production. The studies herein evaluated titanium ion exchanger and examined if the material could be used for the purification of ^225^Ac. Examination of the effects of acid rinses on PCST indicated that even 0.1 M acid, either nitric or hydrochloric, breaks down the material and resulted in the elution of the material (Titanium). This led to the conclusion that acid more dilute than 0.1 M is needed when working with PCST. For the separation of ^225^Ac from radioactive Ra and Th, the optimized method used 18 BV of buffer at pH 5 and 6 BV of buffer at pH 3. This lead to a high recovery of eluted Ac (92.4%) and high radiopurity (99.3%). An ^225^Ac ^229^Th generator can be established based on this separation. Capacity studies of Barium and Thorium on PCST indicated that the material did not have a high enough capacity for a production scale thorium target (≥50 g) of PCST. However, PCST could be used to purify ^225^Ac from smaller production scale ^226^Ra targets (0.3 g).

## Materials and Method

Reagents were used from manufacturer without additional purification: phosphoric acid, fumed silica, titanium isoproproxide and KOH Pellets were purchased from Sigma Aldrich. Sodium hydroxide (98%), nitric acids (70% optima) and trace metal grade hydrochloric acid were purchased from Fisher. La, Ce, Lu ICP standards were purchased form Fluka in 1000 mg/L concentrations. ICP single elemental certified standards of: Th, Ag, Ba, Rh, Ce, and La were purchased from SPEX Certiprep. All solutions were prepared using Milli-Q water and all experiments were conducted at room temperature. All the chemicals used were of analytical reagent grade. Buffers were prepared from previously prepared 0.5 N Sodium Acetate buffer and adjusted with 8 N HCl or 10 M NaOH. Initial and equilibrium pH readings were obtained using a Denver Instruments UB-10 pH/mV meter calibrated at pH 2.0, 4.0 and 7.0. Since Ba chemically behaves similarly to Ra and La is chemically similar to ^225^Ac for some studies Ba and La can be used as surrogates. ^225^Ac radiotracer was supplied by Oak Ridge National Laboratory as a dried sample, and the sample was dissolved in 0.1 M HCl solution prior to use. ^223^Ra and ^227^Th were present in some ^225^Ac samples as a result of the decay of ^227^Ac.

### Synthesis of PCST inorganic ion-exchanger

PCST were synthesized according to published methods^26,27^. Phase and purity was confirmed by powder X-Ray diffraction (patterns collected using a Rigaku MiniFlex II Desktop X-Ray diffractometer sampling at 0.040 degrees at a speed of 1degree/min, starting at 5 degrees and ending at 60 degrees.

### Determination of K_d_ values for different inorganic ion-exchangers

#### ^225^Ac and Th

Experimental solutions, consisting of 20 mg L^−1^ of Th and 3.7 × 10^4^ Bq ^225^Ac in 0.5 M sodium acetate (NaOAc), were adjusted to a pH of 1, 2, 3, 4 or 5 with 10 M NaOH or 69% HNO_3_. Inorganic ion-exchangers HCST (100 ± 0.5 mg) were added to 10 mL of the metal containing solution. The tubes were shaken (using Thermo Scientific compact digital microplate shaker) for 12 hours at room temperature. The tubes were centrifuged (4000 rpm, 2598 RCF) for 4 minutes and the aqueous phases were separated using 0.2 µm micro syringe filter. An aliquot from the aqueous phases was diluted in 2% nitric acid and analysis performed.

#### Rh, Ag, Ba, La, Ce and Th on PCST inorganic ion-exchangers

A solution consisting of: 30 mg L^−1^ of each of Ag, Ba, Rh, Ce, and La; and 150 mg L^−1^ of Th in 0.5 M NaOAc. Ba was used as a surrogate of Ra, and La was used as a surrogate for ^225^Ac as the chemistries are similar. The PCST inorganic ion-exchangers (30 ± 0.3 mg) were added to the 10 mls of the metal containing solution. The samples were processed and analyzed as described in the previous section.

### Analysis and calculations of Batch studies

Actinium activities in the initial, intermediate and final solutions were determined by using a gamma spectrometry (ORTEC) with a calibrated high purity germanium detector^28^. The separation fractions containing ^225^Ac, ^227^Th and ^223^Ra were quantified after 24 hours by gamma spectroscopy at 236 (^227^Th), and 269.6 keV (^223^Ra). After 24 hours the original activity associated with ^213^Bi and ^221^Fr has decayed away (24 hours >10 half-lives of ^213^Bi and ^221^Fr). The presence of ^213^Bi and ^221^Fr in the samples is the result of in growth from ^225^Ac, and activity associated with ^213^Bi and ^221^Fr would be at equilibrium with ^225^Ac. At the time of analysis, ^225^Ac and its daughters (specifically ^221^Fr and ^213^Bi) were at equilibrium with ^225^Ac, and the gamma peak at 440 KeV for Bi-213 was used to quantify ^225^Ac. The 218 KeV gamma peak for ^221^Fr was used to quantify ^225^Ac and similar results were obtained.

The concentration of thorium and other metals were measured using ICP-OES (Perkin Elmer Optima 7300 DV spectrometer), and the instrument was calibrated according to published methods^28^. The wavelength (λ) used for thorium, barium, lanthanum, cerium, rhodium, silver and titanium analysis were 283.73, 233.527, 408.672, 413.764, 343.489, 338.289 and 334.94 nm respectively.

K_d_ values were calculated based on the following equation:

$${K}_{d}=[({C}_{i}-{C}_{f})/{C}_{f}]V/g$$

Where C_i_ = Stock concentration metal, C_f_ = Final concentration metal, V = Volume of stock solution, and g = Measured weight of ion-exchanger. K_d_ values were plotted versus pH, and the standard deviation of triplicate samples was calculated.

### Column studies

#### Column Preparation Method

The inorganic ion exchange material was sifted through >50, 50–100, 100–200, 200–400 and <400 µm mesh sifter. For all column studies the particles between 100 and 200 µm in size were used and approximately 1 gram of material was added to 20 mL 0.5 M ammonium acetate at pH 5. The sample was mixed vigorously then the mixture was allowed to settle before decanting the supernatant. This was repeated three times to remove any small residual particles that impeded column flow and caused poor flow rates. Then the inorganic ion exchange material was used to prepare columns with 0.4–0.5 mL bed volumes. The columns were rinsed with 20 BV of 0.5 M ammonium acetate at pH 5 prior to use, and the pH of the solution eluting the column was at pH 5. Columns were timed to monitor the flow rate.

#### Acid Study of PCST

Two 0.5 mL BV columns of poorly crystalline silicotitanate (PCST) were prepared in 0.5 M ammonium acetate at pH 5. One column was rinsed with 10 mL of 0.5 M ammonium acetate at pHs 5, 3, and 1. Then the column was rinsed with: 4 × 3 mL of each of the following concentrations of nitric acid: 0.1 M, 1 M, 2 M and 3 M. A 500 microL aliquoted of each rinse solution was diluted with 4.5 mLs of 2% Nitric Acid to make an ICP-OES sample, and the amount of titanium was quantified. The experiment was repeated with the second column with the exception hydrochloric acid was used instead of nitric acid.

#### Capacity studies to Evaluate resin for purification of ^225^Ac from production targets or isotope generators

Barium: A 0.4 mL column of PCST was prepared in 0.5 M ammonium acetate at pH 5. A barium solution was prepared by evaporated 25 mg of Ba metal ICP standard and resuspended in 5 mL of 0.5 M ammonium acetate buffer at pH 5. The Ba solution was loaded on the PCST column and the column was rinsed with 3 × 10 mLs of 0.5 M ammonium acetate at pH 5. The amount of barium in the load and rinse solution was quantified by ICP-OES. **Thorium:** The study was repeated for thorium with the exception that a 0.5 mL PCST column was used and the load solution contained 100 mg of thorium.

#### ^225^Ac, ^227^Th, ^223^Ra Separation

pH Elution with either PCST ion exchanger: A 0.5 mL PCST column was prepared in 0.5 M ammonium acetate at pH 5, and a 10 mL load solution of the buffer was prepared with tracer quantities of ^225^Ac (4.00 × 10^5^ Bq), ^227^Th (8.136 × 10^4^ Bq) and ^223^Ra (2.886 × 10^4^ Bq). A HPGE counting sample was prepared by diluting 500 µL of the prepared load solution to 3 mL. The remaining load solution was added to the PCST column. Then the column was rinsed with 3 mL of 0.5 M ammonium acetate at each of the following pH values: 5, 4.5, 4, 3.5, 3, 2.5, 2, 1.5, and 1. The radioactivity in the load and rinse samples were quantified by HPGE analysis and the titanium was quantified in the samples by ICP-OES analysis. The ICP-OES samples were prepared by diluting the 3 mL fractions to 5 mL’s with 2% nitric acid.

Optimized Elution method: A 0.5 mL PCST column and a solution containing ^225^Ac, ^227^Th, and ^223^Ra in 0.5 M ammonium acetate at pH was prepared as described above. The previous experiment was repeated with the following rinse sequence of 0.5 M ammonium acetate: 3 × 3 mL at pH 5, 2 × 3 mL at pH 3, and 2 × 3 mL at pH 1. Radioactivity in the fractions was determined by HPGE analysis and ICP-OES was conducted on one of the pH 5 and 3 rinses and both pH 1 rinses.

## Supplementary information

Supplementary Information

## Data Availability

All data generated or analyzed during this study are included in this published article (and its Supplementary Information files).
