# Supplementary material for: The application of poorly crystalline silicotitanate in production of 225Ac
Source: Sci Rep. 2019 Aug 14;9:11808. doi: 10.1038/s41598-019-48021-7 (PMC6694158; doi:10.1038/s41598-019-48021-7)
Supplement: Supplementary file 1 — Supplementary Information [file 41598_2019_48021_MOESM1_ESM.docx]

**The application of poorly crystalline silicotitanate in production of ^225^Ac**

J. Fitzsimmons^a*^, A. Abraham^b^, D. Catalano^c^, A. Younes^a^, C. S. Cutler^a^, D. Medvedev^a^,

^a^Medical Isotope Research & Production Laboratory, Collider-Accelerator Division, Brookhaven National Laboratory, NY, 11973, USA

^b^ Chemistry Department, Stony Brook University, Stony Brook, NY 11794

^c^ Biochemistry Department, Stony Brook University, Stony Brook, NY 11794

^*^corresponding author; phone: 6313444453 (JF), e-mail: [jfitzsimmons@bnl.gov](mailto:jfitzsimmons@bnl.gov),

**Kd values for ^225^Ac and Th on HCST ion exchanger**

Supplementary Figure S1. Distribution coefficients (Kd) of Ac (A) and Th (B) on HCST ion exchanger at pH values from 1 to 5 in 0.5 M ammonium acetate

**Ba-La Separation:**

A 0.5 mL BV column of PCST was prepared as described in the manuscript.

Then 0.5 ml of both Ba and La ICP standards (1,000) was evaporated and then re-suspended in 10 mL of 0.5 M ammonium acetate at pH 5. An ICP-OES sample was prepared from 500 µL of the solution, and the remainder was loaded on the PCST column. The column was rinsed with 20 BV of 0.5 M ammonium acetate at pH 5 and a fraction was collected every 4 BV for a total of 5 rinses. Next, the column was rinsed with 20 BV of 0.5 M ammonium acetate at pH 1, to elute. Again, a fraction was collected every 4 BV for a total of 5 elutions. ICP-OES samples were prepared by diluting 500 µL of each fraction to 5 ml with 2% Nitric Acid.

**Supplementary Figure S2.** Elution profile of Ba and La on PCST resin from 0.5 ammonium acetate at pH 5 or pH1. La was eluted in the pH 5 buffer and combining the load and rinses 1-3 recovered 85% of the La with minimal Ba.

**Studies to Optimize the Elution profile of PCST with ^225^Ac ,^227^Th, ^223^Ra**

A PCST column and a solution containing ^225^Ac, ^227^Th, ^223^Ra was prepared as described in the manuscript. The column was rinsed with 6 BV of 0.5 M ammonium acetate at both pH 5 and 3, and 12 BV at pH 1, and each fraction was analyzed by high purity germanium detector. The experiment was performed in duplicate. Duplicate separation of decayed ^225^Ac solution containing ^223^Ra and ^227^Th using a PCST column. Method based on results gathered in the separation shown in Figure 4. This data indicates the necessity of additional rinses at pH 5 to avoid the elution of residual ^225^Ac at pH 1; as well as the necessity of additional rinses at pH 1 to elute the majority of ^223^Ra retained on the column.

**Supplementary** **Figure S3.** Elution profile of ^225^Ac, ^223^Ra and ^227^Th on PCST columns with 0.5 M ammonium acetate at pH 5, 3, and 1. The ^225^Ac eluted in load and pH 5 and 3 rinses, but ^227^Th eluted at pH 5 and 1, and ^223^Ra eluted in the pH 1 solution and was retained on the column.

**Column studies ^225^Ac, Th, Ag, Ba, Rh, Ce, La**

A metal containing solution was prepared using 30mg L^-1^ of each of: Ag, Ba, Rh, Ce and La; 150mg L^-1^ of Th and 1µCi of ^225^Ac in 0.5 M sodium acetate at pH 2. A PCST inorganic ion-exchanger column (1 ml bed volumes) was prepared and rinsed with 20 mls of 0.5M sodium acetate buffer solution at pH 2.09 and 5, respectively. The PCST column was loaded with 5 mLs of the metal containing solution, and the column was rinsed with 0.5M NaOAc buffer solution (pH = 2). The eluate was fractionated into 3 mL increments, and the retained metals were eluted with 3 M nitric acid. The metals in the fractions were quantified by gamma spectroscopy or ICP-OES, and the eluted ^225^Ac, were pooled and the pH was adjusted to 5.


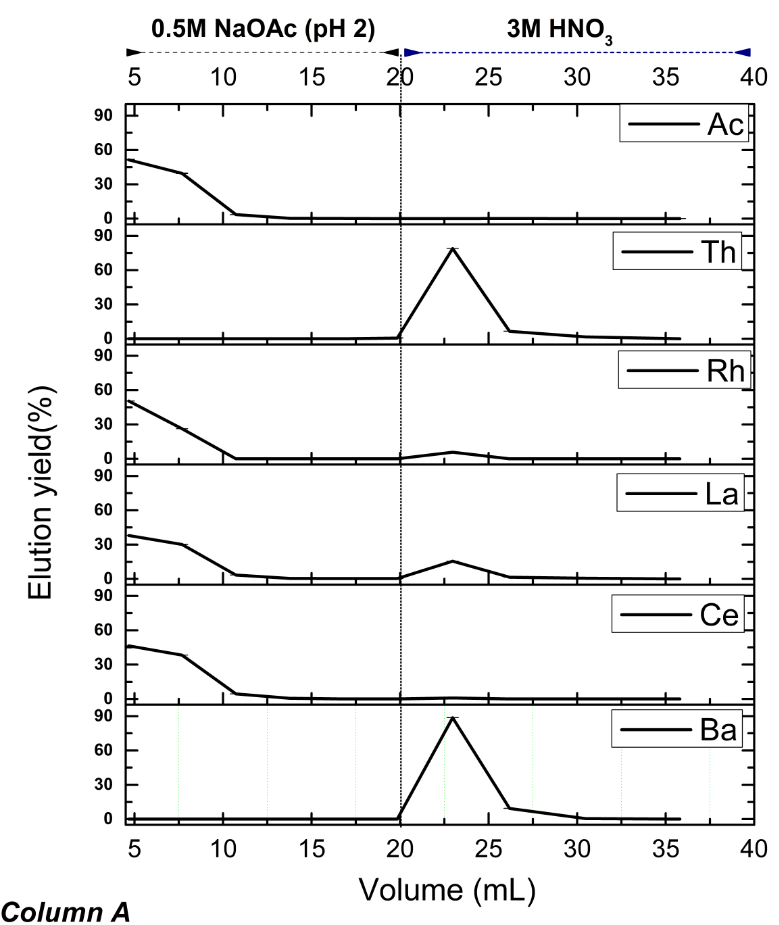


**Supplementary Figure S4.** Elution profiles of ^225^Ac, Th, Rh, La, Ce and Ba on poorly crystalline silicotitanate (PCST) ion exchanger in 0.5 M NaOAc solution at pH 2.
